# Supplementary material for: CD31 signaling promotes the detachment at the uropod of extravasating neutrophils allowing their migration to sites of inflammation
Source: eLife. 2023 Aug 7;12:e84752. doi: 10.7554/eLife.84752 (PMC10431918; doi:10.7554/eLife.84752)
Supplement: Figure 3—figure supplement 1—source data 2. [file elife-84752-fig3-figsupp1-data2.pdf]

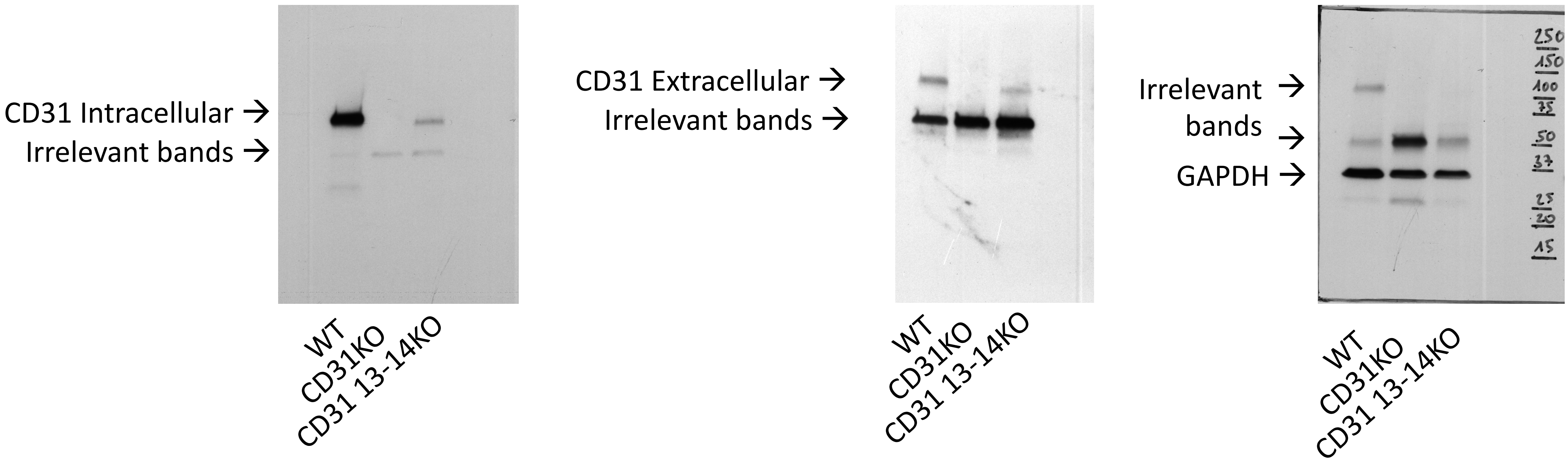
CD31 Intracellular →
Irrelevant bands →
WT
CD31KO
CD31 13-14KO
CD31 Extracellular →
Irrelevant bands →
WT
CD31KO
CD31 13-14KO
Irrelevant bands →
→
GAPDH →
250
150
100
75
50
37
25
20
15
WT
CD31KO
CD31 13-14KO
