# Supplementary figures and images for: CD31 signaling promotes the detachment at the uropod of extravasating neutrophils allowing their migration to sites of inflammation
Source: eLife. 2023 Aug 7;12:e84752. doi: 10.7554/eLife.84752 (PMC10431918; doi:10.7554/eLife.84752)

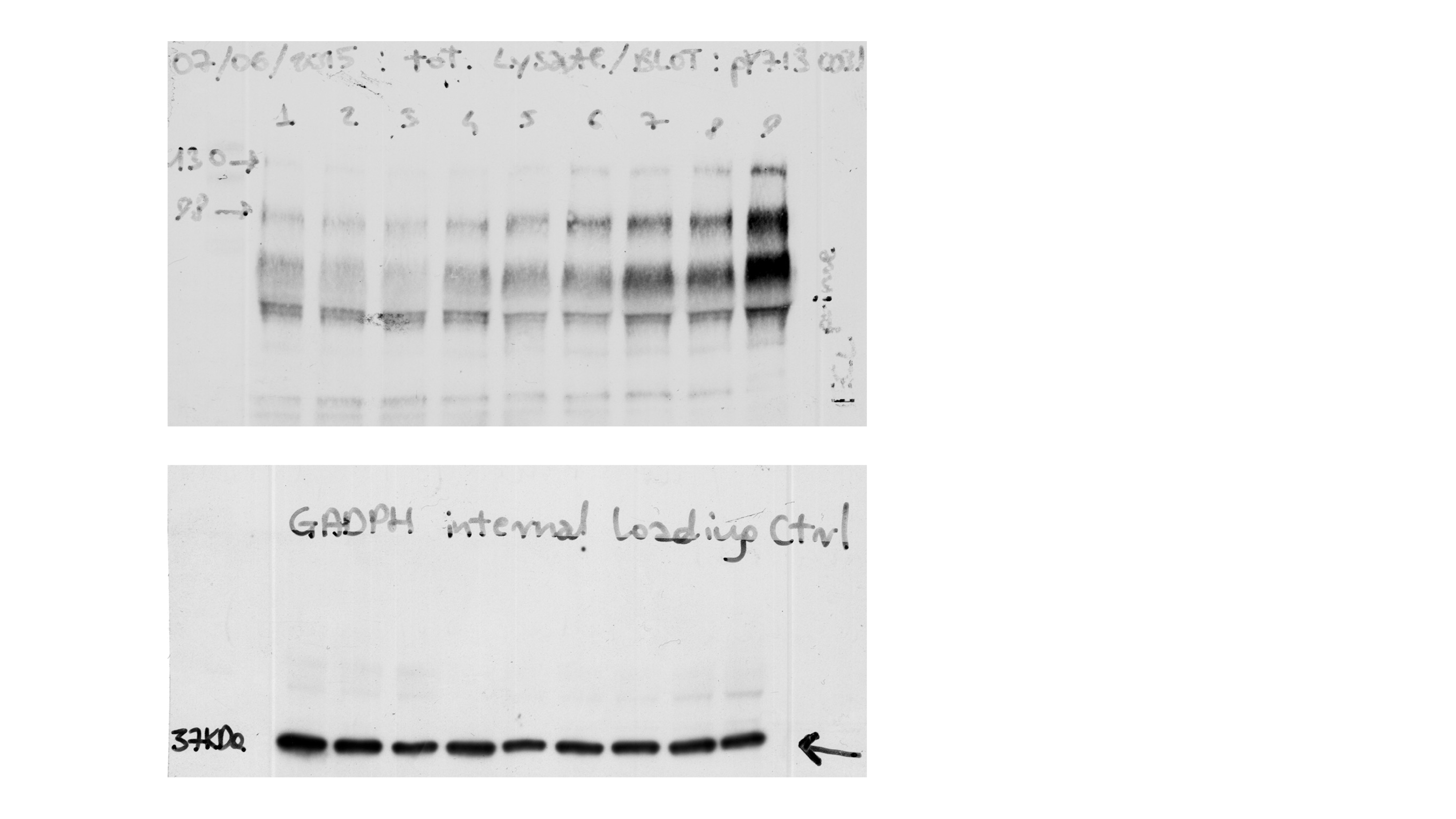

Supplement: Figure 4—figure supplement 1—source data 2. [file elife-84752-fig4-figsupp1-data2.tif]
